# CD8αα^+^T cells exert a pro‐inflammatory role in patients with psoriasis

**DOI:** 10.1002/ski2.64

**Published:** 2021-11-16

**Authors:** Y. Y. Zhang, Y. T. Lin, L. Wang, X. W. Sun, E. L. Dang, K. Xue, W. G. Zhang, K. M. Zhang, G. Wang, B. Li

**Affiliations:** ^1^ Department of Dermatology Xijing Hospital Fourth Military Medical University Xi'an China; ^2^ Institute of Dermatology Taiyuan City Central Hospital Shanxi Key Laboratory for Immunological Dermatosis Taiyuan China

## Abstract

**Background:**

Psoriasis is a common chronic inflammatory disease caused by excessive activation of CD4^+^T cells, including Th17, Th1 and Th22. The role of CD8^+^T cells in psoriasis pathogenesis remains poorly understood.

**Aim:**

To identify the phenotype of CD8^+^T cells in patients with psoriasis and to investigate its role in the formation of lesions.

**Methods:**

The phenotype of CD8^+^T cells in psoriatic lesions was detected by immunofluorescence staining. Flow cytometry was performed to detect their phenotype in peripheral blood. Thereafter, coculture of CD8αα^+^T cells with autogenous CD4^+^T cells was performed to investigate the function of CD8αα^+^T cells in patients with psoriasis. Finally, pro‐inflammatory factors produced by CD8αα^+^T cells were examined by immunofluorescence staining and flow cytometry.

**Results:**

Compared to the CD8αβ^+^T cells, CD8αα^+^T cell infiltration in psoriatic lesions markedly increased. Moreover, epidermal CD8αα^+^T cells exhibited tissue‐resident memory T cells (T_RM_) phenotypes and dermal CD8αα^+^T cells exhibited effector memory (T_EM_) phenotypes in psoriatic lesions. Additionally, we found that CD8αα^+^T cells from patients with psoriasis did not express the markers of regulatory T cells and could promote the proliferation of CD4^+^T effector cells and produce interleukin‐17 and interferon‐γ.

**Conclusions:**

Our findings demonstrate that CD8αα^+^T cells contribute to the pathogenesis of psoriasis by producing pro‐inflammatory factors.

## Graphical abstract

We identified a new subset of CD8^+^T cells, CD8αα^+^TRM cells, which contribute to psoriasis by producing pro‐inflammatory cytokines.

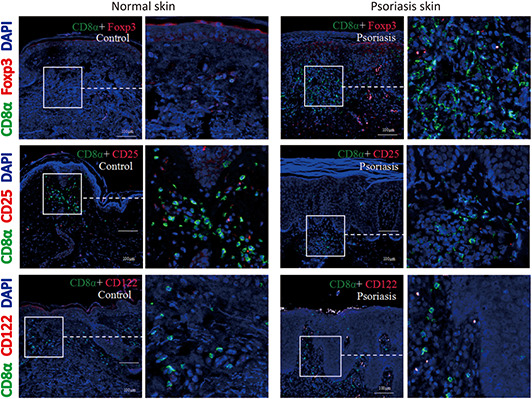

1

**What is already known about this topic?**

- Psoriasis is a common chronic inflammatory disease. The role of CD8^+^T cells in psoriasis pathogenesis remains poorly understood.

**What does this study add?**

- CD8αα^+^T cells infiltrate psoriatic lesions increasingly compared with CD8αβ^+^T. Epidermal CD8αα^+^T cells exhibit tissue‐resident memory phenotypes, and dermal CD8αα^+^T cells exhibit effector memory phenotypes. CD8αα^+^T cells exhibit a pro‐inflammatory role in psoriasis.

## 2 INTRODUCTION

Psoriasis is a common T cell‐mediated chronic inflammatory disorder. It is generally accepted that excessive activation of innate and adaptive immune response is critical for psoriasis pathogenesis via production of interleukin‐17 (IL‐17), interferon‐γ (IFN‐γ) and tumour necrosis factor‐α (TNF‐α).^1^, ^2^ Recent studies have shown that in addition to CD4^+^T cells, γδT cells, dendritic cells, neutrophils and CD8^+^T cells are also found to produce pro‐inflammatory factors in patients with psoriasis.^3^, ^4^, ^5^

CD4 and CD8 are expressed on the surface of T cells as co‐receptors to enhance sensitivity to antigens. CD8 is different from CD4 in that it functions as a dimer, including homodimer CD8αα and heterodimer CD8αβ. CD8αα has been found in different immune cells, including T cells, dendritic cells and natural killing cells.^6^, ^7^ CD8αβ is specifically expressed in T cells, which is called cytotoxic T cells. CD8αβ is a superior T‐cell co‐receptor that recruits T‐cell receptors (TCRs) to lipid rafts and enhances the interaction of TCR‐CD3 activation complexes with the major histocompatibility complex (MHC) class I. However, CD8αα is excluded from lipid rafts and negatively regulates TCR activation by disrupting lipid rafts and sequestering signalling molecules required for TCR‐initiated downstream signalling.^8^, ^9^

Previous studies have shown that CD8αα^+^T cells are found in the gut epithelium and can interact with intestinal epithelial cells by CD8αα binding to Thymus leukaemia (TL, non‐classical MHC class I molecules). CD8αα^+^T cells function as regulatory T cells in the intestinal mucosa, although they do not express Foxp3.^10^, ^11^ Additionally, unlike CD8αβ^+^T cells, TCR of CD8αα^+^T cells is oligoclonal and recognizes peptide fragments from TCRs or MHC. Thus, CD8αα^+^T cells exert a suppressive role by targeting effector T cells or antigen‐presenting cells.^12^ However, whether CD8αα^+^T cells contribute to the pathogenesis of psoriasis has not been clarified. In our study, we investigate the phenotypes of CD8αα^+^T cells in patients with psoriasis and its role in lesion formation.

## 3 MATERIALS AND METHODS

### 3.1 Patients and samples

Blood and skin samples were obtained from the tissue bank of Department of Dermatology, Xijing Hospital of the Fourth Military Medical University. All patients with psoriasis were diagnosed clinically and pathologically in the progressive stage with mild‐to‐moderate psoriasis (psoriasis area and severity index, PASI ≥ 5) or severe psoriasis (PASI ≥ 10). These patients did not receive any topical treatment for a minimum of 1 month and were naïve to photo(chemo)therapy or systemic treatments for psoriasis. All patients and controls were otherwise healthy and did not use any other systemic medications. Twenty‐millilitre blood samples were collected from each patient or who was a healthy control. Normal skin and psoriatic lesions were obtained from patients via biopsy (Table S1). This study was approved by the ethical committee of Xijing Hospital of the Fourth Military Medical University. All healthy volunteers and patients with psoriasis agreed to participate in this study and provided written informed consent.

### 3.2 Immunofluorescence analysis

Immunofluorescence was performed on paraffin‐embedded 5‐μm sections of skin samples. Following paraffin removal, epitope retrieval and blocking in phosphate buffered saline/4% bovine serum albumin, sections were incubated with primary antibodies for 16 h at 4°C. Thereafter, secondary antibodies were added and incubated for 1 h at room temperature in the dark. Nuclei were stained with Hoechst (Beyotime, at a dilution of 1:1000) for 10 min at room temperature in the dark. Immunofluorescence staining was analysed using an OLYMPUS FV10 confocal laser scanning microscope 510 system (Carl Zeiss AB). Primary and secondary antibodies are listed in Table S2.

### 3.3 Flow cytometry

To detect cytokine production, fresh peripheral blood mononuclear cells (PBMCs) were incubated with lipopolysaccharides (10 μg/ml; Sigma) for 12 h to activate monocytes exhibiting antigen presentation, followed by a cell stimulation cocktail (Phorbol‐12‐myristate‐13‐acetate, 50 ng/ml; ionomycin, 1 μg/ml; Golgi Stop 0.2 μL; eBioscience) for 4 h to amplify the immune response and maintain cytokines intracellularly. Then, PBMCs were labelled with anti‐human CD3‐PE/CY7, anti‐human CD8α‐PE/CY5 and anti‐human CD8β‐eFluor 660 for 30 min at 4°C in the dark. Fixation/permeabilization solution and permeabilization buffer (eBioscience) were used to fix and permeabilize PBMCs according to the manufacturer's instructions. Finally, they were stained with anti‐human IL‐17A‐PE, and anti‐human IFN‐γ‐PE for 30 min at 4°C in the dark, and analysed by flow cytometry (fluorescence‐activated cell sorting [FACS], Calibur, BD). All the antibodies are listed in Table S2.

### 3.4 T cell isolation and sorting

Peripheral blood mononuclear cells were separated using Ficoll‐Hypaque (Dakewei). Cells were labelled with anti‐human CD3‐PECY7, anti‐human CD4‐APC, anti‐human CD8α‐PE and anti‐human CD8β‐eFluor 660 for 30 min at 4°C in the dark. Thereafter, CD3^+^CD4^+^Tresp cells, CD3^+^CD8α^+^CD8β^+^T cells and CD3^+^CD8α^+^CD8β^−^T cells were purified using FACS (Moflo XDP, Beckman) at purity >95%. Isolated cells were subsequently cultured in RPMI 1640 supplemented with 10% foetal bovine serum (Gibco).

### 3.5 In vitro function assay of CD8αα^+^T cells

To investigate the suppressive function of CD8αα^+^T cells, FACS‐sorted CD4^+^T cells from healthy subjects and patients with psoriasis (5 × 10^4^ per well) were labelled with carboxyfluorescein succinimidyl ester (CFSE) and subsequently cocultured separately with CD8αα^+^T cells (5 × 10^4^ per well) isolated from the same subject in 96‐well U‐bottom plates. Thereafter, dynabeads human T‐activator CD3/CD28 at a bead‐to‐cell ratio of 1:1 and rIL‐2 (3 μg/ml) were added to cocultures and incubated for 96 h at 37°C. The proliferation of CD4^+^T cells was then analysed using flow cytometry based on the CFSE dilution.

### 3.6 Statistical analysis

Data analysis was performed using the GraphPad Prism version 7.0 software (GraphPad Software). Statistically significant differences between two groups were determined by two‐tailed *t*‐tests. Values of *p* less than 0.05 were considered statistically significant. Data are expressed as the mean standard error.

## 4 RESULTS

### 4.1 Increased CD8αα^+^T cells located in psoriatic lesions

**FIGURE 1:**
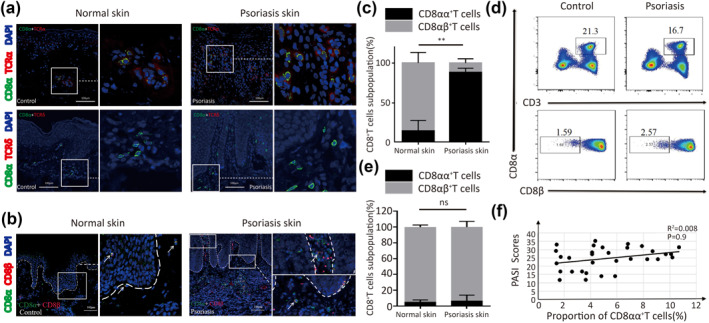
(a–f) CD8αα^+^T cells increasingly infiltrate into psoriatic lesions. Representative immunofluorescence staining of CD8α co‐expressed with (a) TCRα, TCRδ and (b) CD8β in psoriatic lesions and normal skin. (c) Proportion of CD8αβ^+^T and CD8αα^+^T cells determined by analysis of immunofluorescence staining (*n* = 8). (d and e) Frequency of circulating CD8αβ^+^T and CD8αα^+^T cells (bottom) in gated CD3^+^CD8^+^T cells (up) from patients with psoriasis (*n* = 32) and healthy controls (*n* = 16). (f) Frequency correlation of circulating CD8αα^+^T cells with psoriasis area and severity index (PASI) scores of patients with psoriasis (*n* = 32). **p < 0.01

Immunofluorescence staining of CD8α with markers for T cells was performed to identify whether CD8^+^T cells or other CD8α^+^ immune cells infiltrated in psoriatic lesions. The results showed that CD8α co‐expressed with CD3 and TCRα but not with TCRδ, CD11c or CD56 (Figure S1 and Figure 1a), which suggested that CD8^+^T cells, and not γδT cells, dendritic cells (DC) or natural killer (NK) cells, infiltrated psoriatic lesions.

To investigate the subset of CD8^+^T cells in psoriatic lesions, we detected CD8α and CD8β expression by immunofluorescence staining. We found that the proportion of CD8αα^+^T cells markedly increased in psoriatic lesions (88.48% ± 7.39%, *p* = 0.0092) and infiltrated both the dermis and epidermis. However, in normal skin, we found that CD8αβ^+^T cells were predominantly scattered throughout the derma‐epidermal junction (Figure 1b,c).

Next, we investigated the frequency of circulating CD8αα^+^T cells in patients with psoriasis using flow cytometry. As shown in Figure 1d, the frequency of CD8αα^+^T cells was comparable in patients with psoriasis and healthy controls (Figure 1e, 6.54% ± 1.81% vs. 5.48% ± 1.04%, *p* > 0.05). There was no correlation between frequency of psoriatic circulating CD8αα^+^T cells and the PASI score (Figure 1f, *R* ^2^ = 0.008, *p* = 0.9). Thus, these data indicated that lesional CD8αα^+^T cells, but not CD8αβ^+^T cells, infiltrated in the psoriatic lesions.

### 4.2 Epidermal CD8αα^+^T cells exhibit tissue‐resident memory T cells (T_RM_) phenotypes and dermal CD8αα^+^T cells exhibit effector memory (T_EM_) phenotypes in psoriatic lesions

**FIGURE 2:**
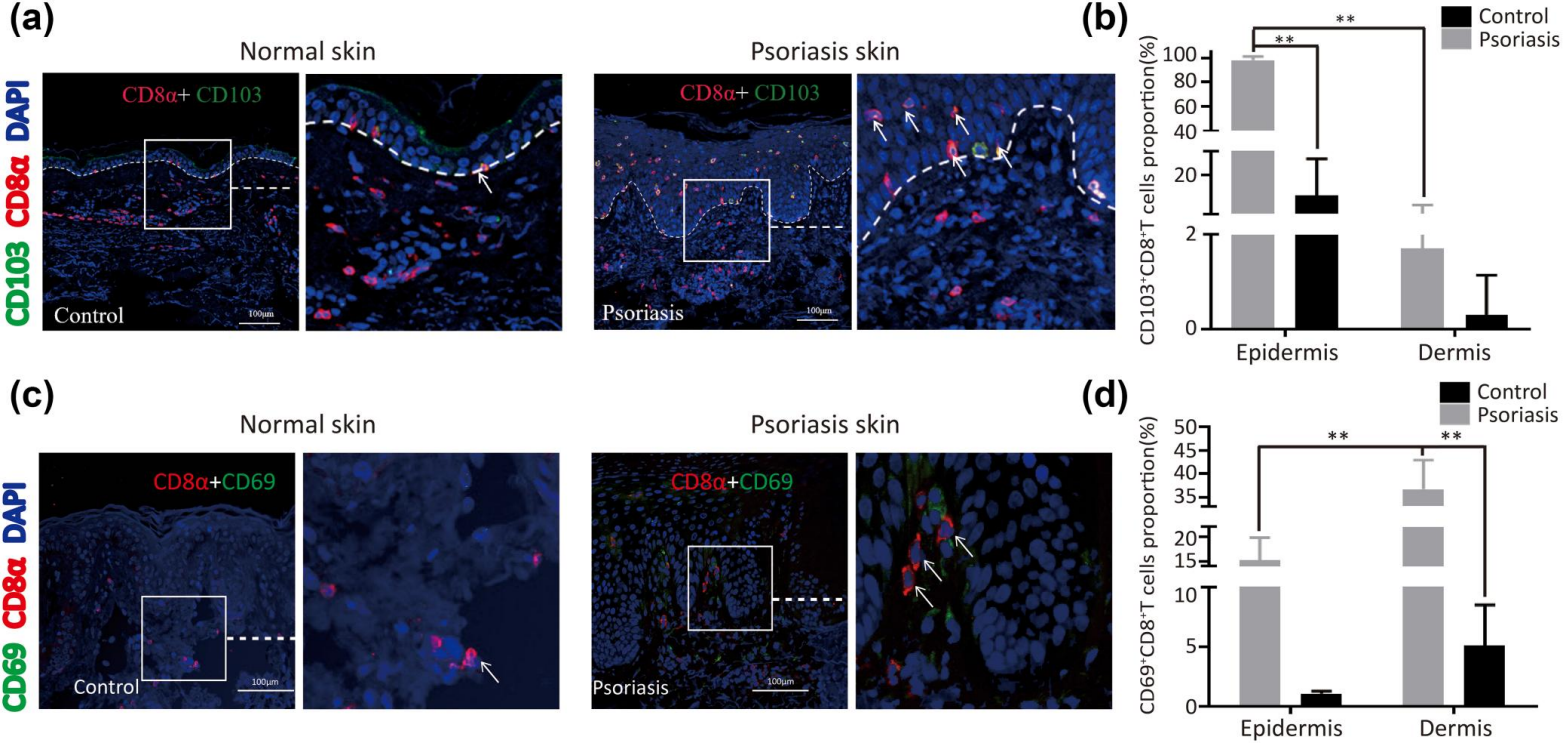
(a–d) Epidermal CD8αα^+^T cells exhibit T_RM_ phenotypes. Representative immunofluorescence staining of (a) CD103 and (c) CD69 expressed in CD8αα^+^T cells of psoriatic lesions and normal skin. Proportion of (b) CD103^+^CD8^+^T_RM_ and (d) CD69^+^CD8^+^T_RM_ cells determined by analysis of immunofluorescence staining (*n* = 8). **p < 0.01

We investigated CD8αα^+^T cell phenotypes in patients with psoriasis using the immunofluorescence assay. We found that approximately 90% of the CD8αα^+^T cells did not express CD45RA and CCR7 in psoriatic lesions. In normal skin, CD8^+^T cells did not express CCR7 but partially expressed CD45RA (Figure S2a,b). CD103 and CD69, identified as tissue‐resident memory markers, were expressed in T_RM_ cells. Notably, we found that more than 95% of the CD8αα^+^T cells expressed CD103 in the epidermis of patients with psoriasis, but approximately 1.7% of the CD8αα^+^T cells expressed CD103 in the dermis (Figure 2a,b). Moreover, approximately 17.2% of the CD8αα^+^T cells expressed CD69 in the epidermis of patients with psoriasis, but approximately 38.3% of the CD8αα^+^T cells expressed CD69 in the dermis (Figure 2c,d).

Thus, these results indicate that psoriatic epidermal CD8αα^+^T cells were predominantly T_RM_ cells, which exhibited the CD69^−^CD103^+^ phenotype, and psoriatic dermal CD8αα^+^T cells were T_EM_ cells, which exhibited the CD69^+/−^CD103^−^CCR7^−^CD45RA^−^ phenotype.

### 4.3 CD8αα^+^T cells do not exhibit a regulatory role in psoriasis

**FIGURE 3:**
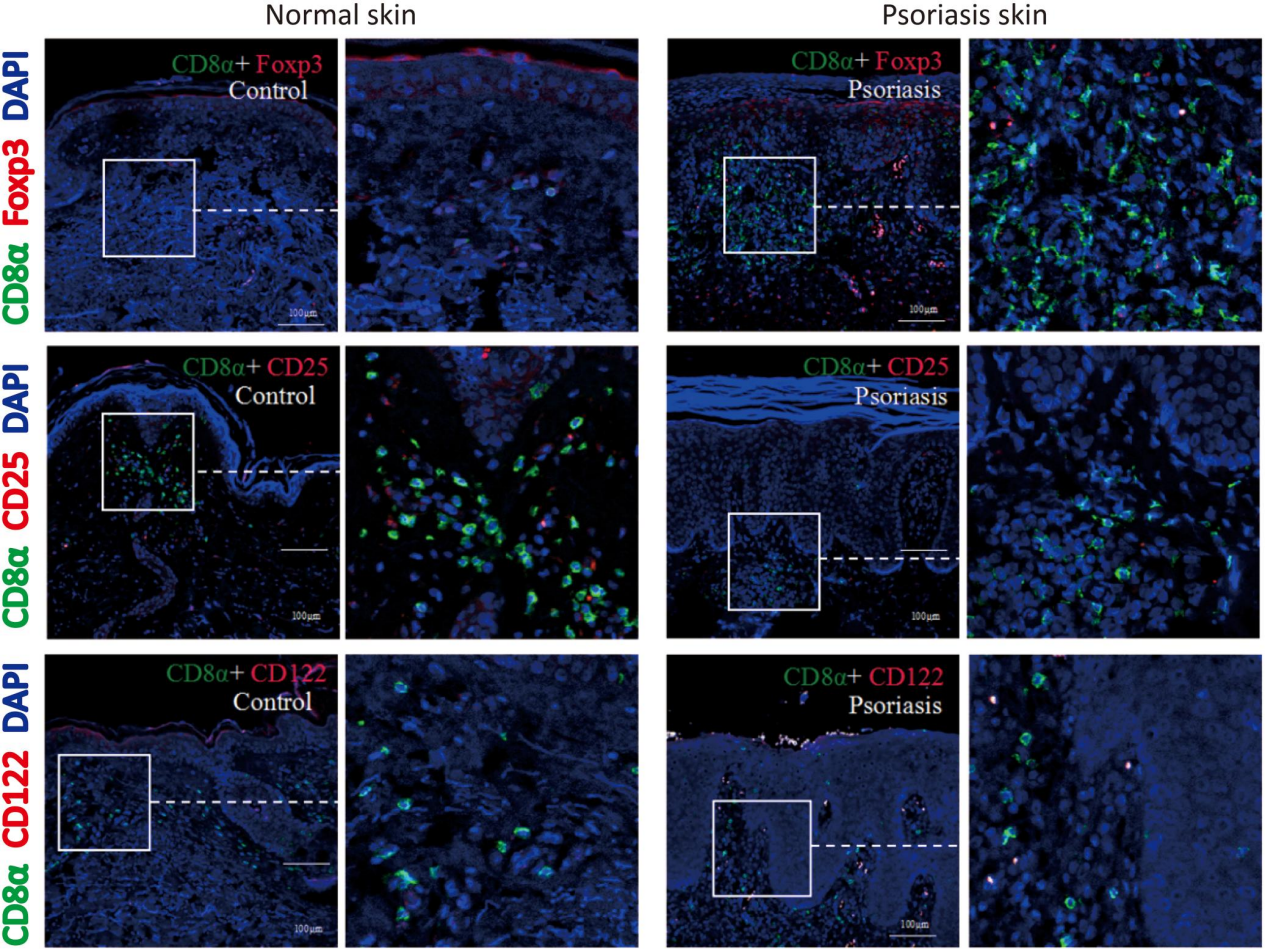
CD8αα^+^T cells do not express markers of Treg cells in psoriatic lesions. Representative immunofluorescence staining of Foxp3, CD25 and CD122 expressed in CD8αα^+^T cells of psoriatic lesions and normal skin

CD8αα^+^T cells expressed Treg cell markers and exhibited a regulatory role in intestinal mucosa. To determine whether CD8αα^+^T cells have a suppressive immune function in psoriasis, we detected the expression of Treg cell markers in psoriatic CD8αα^+^T cells. Immunofluorescence showed that CD8αα^+^T cells expressed little CD122 and did not express Foxp3 and CD25 in psoriatic lesions (Figure 3).

**FIGURE 4:**
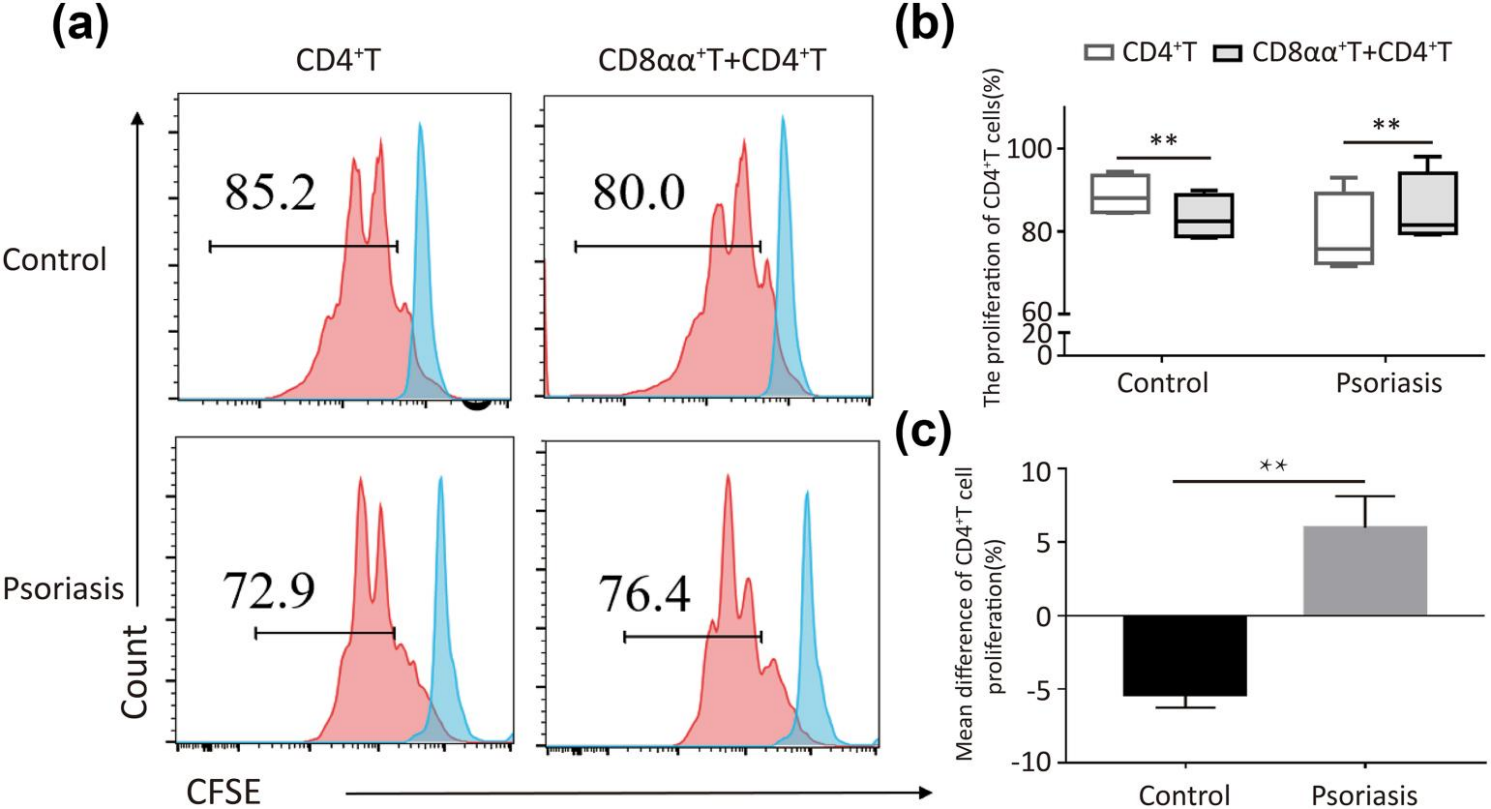
(a–c) CD8αα^+^T cells exhibit a pro‐inflammatory role in patients with psoriasis. (a) CD8αα^+^T cells from patients with psoriasis or healthy controls were cocultured with autogenetic CD4^+^T cells. Proliferation of CD4^+^T cells was detected by flow cytometry, which could reflect the function of CD8αα^+^T cells. (b) Statistical analysis of flow cytometry. (c) Mean difference of CD4^+^T cell proliferation (proportion of proliferated CD4^+^T cells in the coculture group minus that in the group of autogenetic CD4^+^T cells alone, *n* = 5). **p < 0.01

For verification, we used an in vitro lymphocyte coculture model of CD8αα^+^T cells and CD4^+^T cells. We analysed the suppressive function of CD8αα^+^T cells by assessing the proliferation of CD4^+^T cells using flow cytometry. We found that CD8αα^+^T cells enhanced the proliferation of CD4^+^T cells in patients with psoriasis but inhibited its proliferation in healthy controls (Figure 4a–c). These results suggest that CD8αα^+^T cells exhibit a pro‐inflammatory role in psoriasis but not a suppressive function.

### 4.4 CD8αα^+^T cells exhibit pro‐inflammatory activity in psoriasis

**FIGURE 5:**
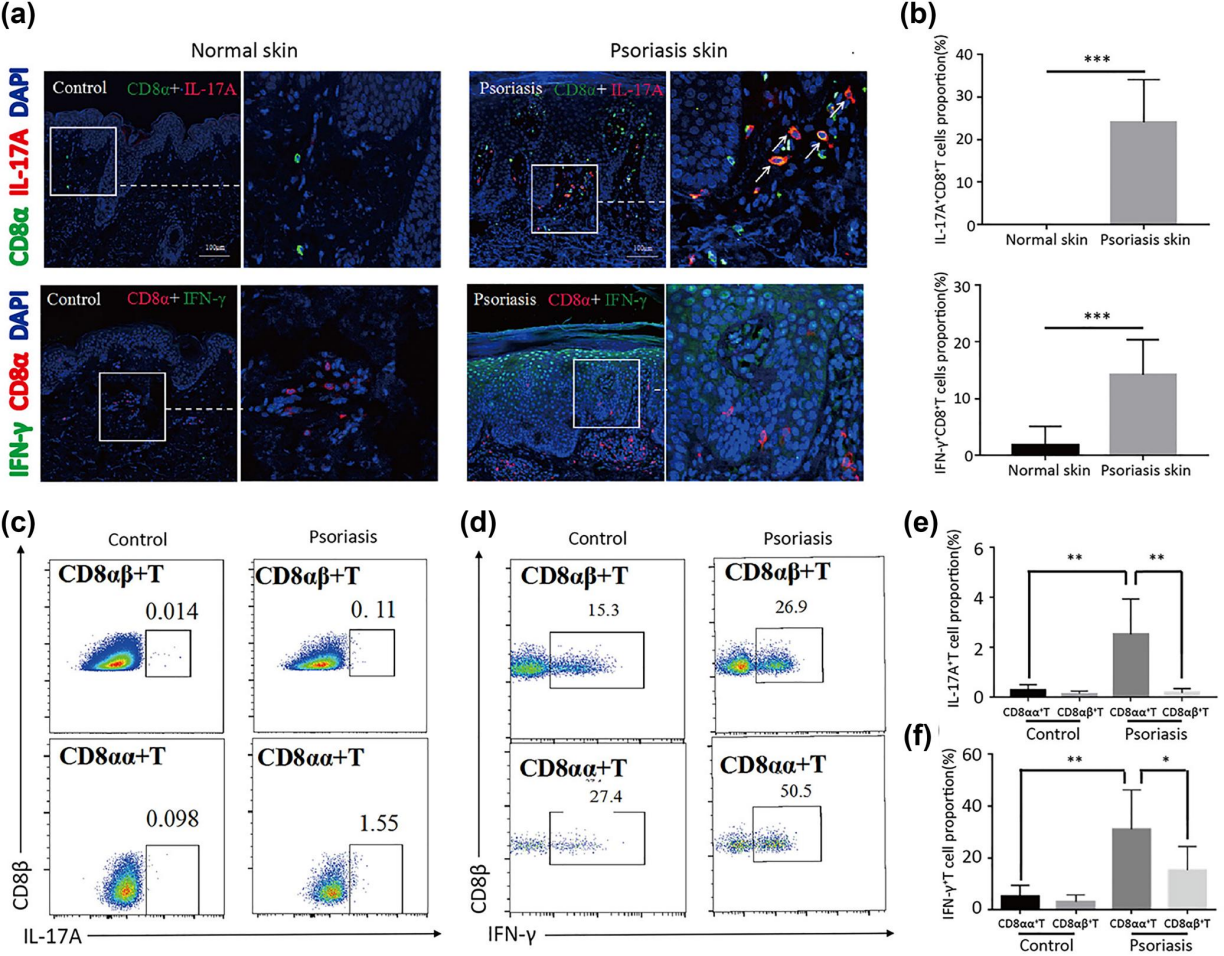
(a–f) CD8αα^+^T cells produce pro‐inflammatory factors in patients with psoriasis. (a) Representative immunofluorescence staining of IL‐17A and interferon‐γ (IFN‐γ) expressed in CD8αα^+^T cells of psoriatic lesions and normal skin. (b) Proportion of IL‐17A^+^ CD8αα^+^T and IFN‐γ^+^CD8αα^+^T cells determined by analysis of immunofluorescence staining (*n* = 8). (c) The expression of IL‐17A and (d) IFN‐γ in circulating CD8αα^+^T cells from patients with psoriasis (*n* = 32) and healthy controls (*n* = 16) by flow cytometry. (e and f) Statistical analysis of flow cytometry. *p < 0.05; **p < 0.01; ***p < 0.001

To further investigate the mechanism of how CD8αα^+^T cells exhibited a pro‐inflammatory function, we detected cytokines produced by CD8αα^+^T cells in psoriasis. We found that CD8αα^+^T cells could produce IL‐17A and IFN‐γ in lesions of patients with psoriasis but did not express these cytokines in normal skin (Figure 5a,b). Additionally, CD8αα^+^T cells from psoriatic peripheral blood produced significantly higher levels of IL‐17A and IFN‐γ than those from healthy controls (Figure 5c–f). Moreover, CD8αα^+^T cells produce higher levels of IL‐17A and IFN‐γ than CD8αβ^+^T cells, both in peripheral blood of patients with psoriasis and healthy controls. Thus, these results suggest that CD8αα^+^T cells exhibit a pro‐inflammatory function by producing IL‐17A and IFN‐γ in psoriasis.

## 5 DISCUSSION

The current study investigated the function of CD8αα^+^T cells in psoriasis. We found that the proportion of CD8αα^+^T cells was markedly increased in psoriatic lesions. In addition, psoriatic epidermal CD8αα^+^T cells were predominantly CD69^−^CD103^+^CCR7^−^CD45RA^−^T_RM_ cells, whereas psoriatic dermal CD8αα^+^T cells were CD69^+/−^CD103^−^CCR7^−^CD45RA^−^T_EM_ cells. Moreover, CD8αα^+^T cells exhibited a pro‐inflammatory role in psoriasis by producing IL‐17A and IFN‐γ. Therefore, our results indicate that CD8αα^+^T_RM_ cells contribute to psoriasis by producing pro‐inflammatory cytokines.

Previous studies have shown that dense infiltration of CD8^+^T cells is found in the psoriatic lesions. CD8^+^T cells have been identified as a potent source of pro‐inflammatory cytokines in psoriasis.^3^, ^13^ However, these studies solely identify CD8^+^T cells by detecting CD8α expression. CD8α is expressed not only on T cells but also on the surface of NK cells, DC and γδT cells. Ovigne et al. previously detected the phenotype of epidermal CD8^+^T cells in psoriatic patients. They isolated epidermal CD8α^+^cells using flow cytometry, which then stimulated to expand with irradiated PBMC, IL‐2 and phytohemagglutinin for 11–14 days. The results showed that most of the expanded CD8α^+^cells were CD8αβ^+^ (93.3%) and TCRαβ^+^ (99.5%) and produce highly heterogeneous levels of IFN‐γ.^14^ Considering the phenotype of CD8^+^T cells may be influenced by culture in vitro, we performed immunofluorescence to directly detect the phenotype of CD8^+^T cells in the lesions of psoriatic patients. We found that the identity of infiltrating CD8^+^ cells was not NK cells, DC or γδT cells but TCRαβ^+^T cells. Furthermore, CD8αα^+^T cells primarily infiltrated psoriatic lesions and not CD8αβ^+^T cells.

Like a subpopulation of Th cells, according to different kinds of cytokine production, CD8^+^T cells can be divided into Tc1, Tc2, Tc17 and Tc22. Additionally, unlike the CD4 oligomer, CD8 can be expressed as a CD8αα homodimer and CD8αβ heterodimer on the surface of CD8^+^T cells.^7^ Although CD8αα andCD8αβ bind similarly to MHC class I molecules, CD8β endows CD8 with co‐receptor function. Indeed, CD8αβ, but not CD8αα, associated with TCR/CD3, strengthens pHMC binding and promotes CD8 association with lipid rafts and p56^lck^ and hence TCR signalling. CD8αα is excluded from lipid rafts and negatively regulates TCR activation.^15^ As we know, CD8αβ expressed on the surface of T cells is also called cytotoxic T cells. However, CD8αα is expressed on T cells, NK cells, DC and γδT cells. CD8αα^+^T cells were first found to be infiltrating the intestine of mouse and contributed to the pathogenesis of intestinal diseases associated with infection and inflammation.^12^ Little is known about its role in human bodies. Zhu et al. found that CD8αα^+^T cells persisted in the genital skin and mucosa at the derma‐epidermal junction to exhibit an immune surveillance role in herpes simplex virus infection.^16^ Consistent with this study, we found markedly increased number of CD8αα^+^T cells in the lesions of psoriasis, and they have a pro‐inflammatory function.

In the mouse model of inflammatory bowel disease, CD8αα^+^T cells were found to play an immunosuppressive role. As mentioned above, CD8αα can negatively regulate TCR activation by interacting with its ligand, TL, which is abundantly expressed by intestinal epithelial cells. Although Foxp3 and CD25 (IL‐2 receptor subunit α) were identified as markers of Treg cells, they are not expressed in CD8αα^+^T cells.^10^, ^11^ However, in the intestine of mice, CD122 (IL‐2/15 receptor subunit β) is found on the surface of CD8αα^+^T cells and is necessary for cell survival. Consistent with previous studies, we found that Foxp3 and CD25 were negatively expressed in CD8αα^+^T cells of patients with psoriasis, and unexpectedly, CD122 was also expressed little.

Furthermore, previous studies have shown the immunoregulatory role of CD8aa^+^T cells in some animal models. Non‐obese diabetic mice are defective in CD8aa^+^T cell generation.^17^ Additionally, transfer of CD8aa^+^T cells can inhibit colitis, induced by the adoptive transfer of CD4 T cells into severe combined immunodeficient mice.^18^ Unlike CD4^+^Treg cells, the mechanisms underlying the immunosuppressive function of CD8αα^+^T cells were that they recognized pathogenic TCR‐derived peptides, which is presented on the MHC‐I Qa‐1 molecule of pathogenic T cells in an activation‐dependent manner.^9^, ^19^, ^20^ Therefore, to further determine whether CD8αα^+^T cells have an immunosuppressive function in psoriasis, we cocultured CD8αα^+^T cells with auto‐CD4^+^T cells. Consistent with previous studies, CD8αα^+^T cells from healthy subjects suppressed the proliferation of auto‐CD4^+^T cells but promoted its proliferation in patients with psoriasis. Thus, our results indicate that CD8αα^+^T cells play a pro‐inflammatory role in psoriasis, instead of an immunosuppressive function.

In mice, CD8αα^+^T cells are identified as T_RM_ cells, which persist in the same sites of intestinal epithelium for a long time, noncirculating and largely maintained independently of circulating populations.^12^ These T_RM_ cells are phenotypically heterogeneous, and there are no perfect markers of tissue residency; the most commonly associated markers are CD69 (c‐type lectin), CD103 (an E‐cadherin receptor), CD49a and CXCR3/6. Additionally, unlike central memory (T_CM_) and T_EM_ cells, T_RM_ cells do not express CCR7 (a lymph node homing receptor) and CD62L (L‐selectin).^21^, ^22^, ^23^ Previous studies have shown that CD103^+^CD8^+^T_RM_ cells and CD69^+^CD8^+^T_RM_ cells infiltrated in psoriatic lesions and non‐lesional psoriatic skin; they are even found in the cleared skin after treatment and could produce IL‐17, IFN‐γ and IL‐22 persistently.^24^, ^25^ Cheuk et al. reported that CD49a^−^CD8^+^T_RM_ cells from psoriatic lesions predominantly generated the IL‐17 response, whereas CD49a^+^CD8^+^T_RM_ cells preferentially produced IFN‐γ and displayed high cytotoxic capacity contributing to vitiligo.^26^ In our study, we found that about 90% of the CD8^+^T cells in psoriatic lesions are of the CD8αα^+^phenotype and identified a new phenotype of T_RM_ cells, CD103^+^CD8αα^+^, in the epidermis of psoriatic lesions. Additionally, CD103^−^CD8αα^+^T cells in the dermis may be not T_RM_ cells. Although CD69 be considered to be marker of T_RM_ cells, it can also be expressed in T effector cells instantaneously. We found that CD69 mainly expressed in CD8αα^+^T_EM_ cells in dermis of psoriasis lesions. Moreover, CD8αα^+^T cells from both epidermis and dermis do not express CCR7 and CD45RA (a naïve T cell marker) in psoriatic lesions. Thus, our results indicate that CD8αα^+^T cells, as a subset of T_RM_ cells in epidermis, contribute to the pathogenesis of psoriasis.

However, there are still some limitations in our study. Because it is difficult to obtain enough skin tissue from patients with psoriasis, we just performed immunofluorescence to identify the phenotype of CD8αα^+^T cells in lesions. Flow cytometry will be better to identify the phenotype of lesional CD8αα^+^T cells. In addition, although we found CD103^+^CD8αα^+^T_RM_ cells infiltrated the epidermis of psoriasis, some of them did not express CD69. Difference of CD69^+^CD103^+^CD8αα^+^T_RM_ cells from CD69^−^CD103^+^CD8αα^+^T_RM_ cells should be further investigated.

## 6 CONCLUSION

In conclusion, our results suggested that CD8αα^+^T cells exhibited an immunosuppressive function in healthy controls and exhibited the tissue‐resident memory phenotype in psoriatic epidermis, playing a pro‐inflammatory role by producing elevated levels of IL‐17A and IFN‐γ. CD8αα^+^T cells in the dermis may redirect from peripheral blood exhibiting the phenotype of T_EM_ cells to contribute to the pathogenesis of psoriasis.

## CONFLICT OF INTEREST

The authors have no potential conflict of interest to declare.

## AUTHOR CONTRIBUTIONS

**Y. Y. Zhang:** Data curation; Formal analysis; Investigation; Methodology; Project administration. **Y. T. Lin:** Data curation; Formal analysis; Investigation; Methodology; Writing – original draft. **L. Wang:** Formal analysis; Investigation; Project administration; Resources; Writing – original draft. **X. W. Sun:** Data curation; Formal analysis; Methodology; Resources. **E. L. Dang:** Methodology; Project administration; Resources; Software. **K. Xue:** Formal analysis; Methodology; Software. **W. G. Zhang:** Funding acquisition; Methodology; Project administration. **K. M. Zhang:** Conceptualization; Project administration; Resources; Software. **G. Wang:** Conceptualization; Project administration; Writing – review & editing. **B. Li:** Conceptualization; Project administration; Supervision; Writing – original draft; Writing – review & editing.

## Supporting information

Supplementary Material 1

Supplementary Material 2

Supplementary Material 3

Supplementary Material 4

Supplementary Material 5
