# Supplementary material for: CD8αα+T cells exert a pro‐inflammatory role in patients with psoriasis
Source: Skin Health Dis. 2021 Nov 16;1(4):e64. doi: 10.1002/ski2.64 (PMC9060015; doi:10.1002/ski2.64)
Supplement: Supplementary file 1 — Supplementary Material 1 [file SKI2-1-e64-s005.docx]

**Figure S1. The phenotypes of CD8+T cells in psoriatic lesions.** Representative immunofluorescence staining of CD8α co-expressed with CD3, CD11c, CD56.

**Figure S2. (a-d) Dermal CD8αα^+^T cells exhibit T_EM_ phenotypes in psoriatic lesions.** (a) Representative immunofluorescence staining of CD45RA and CCR7 in CD8αα^+^T cells of psoriatic lesions and normal skin. (b) Proportion of CD45RA^-^CCR7^-^T_EM_ and (d) CD45RA^+^CCR7^-^T cells determined by analysis of immunofluorescence staining. ***P < 0.01.*

**Table S1. Information of patients with psoriasis and healthy controls who contributed skin or blood samples.**

**Table S2. Information of Antibody used for immunoflurescene and Flow cytometry.**
