# Supplementary figures and images for: CD8αα+T cells exert a pro‐inflammatory role in patients with psoriasis
Source: Skin Health Dis. 2021 Nov 16;1(4):e64. doi: 10.1002/ski2.64 (PMC9060015; doi:10.1002/ski2.64)

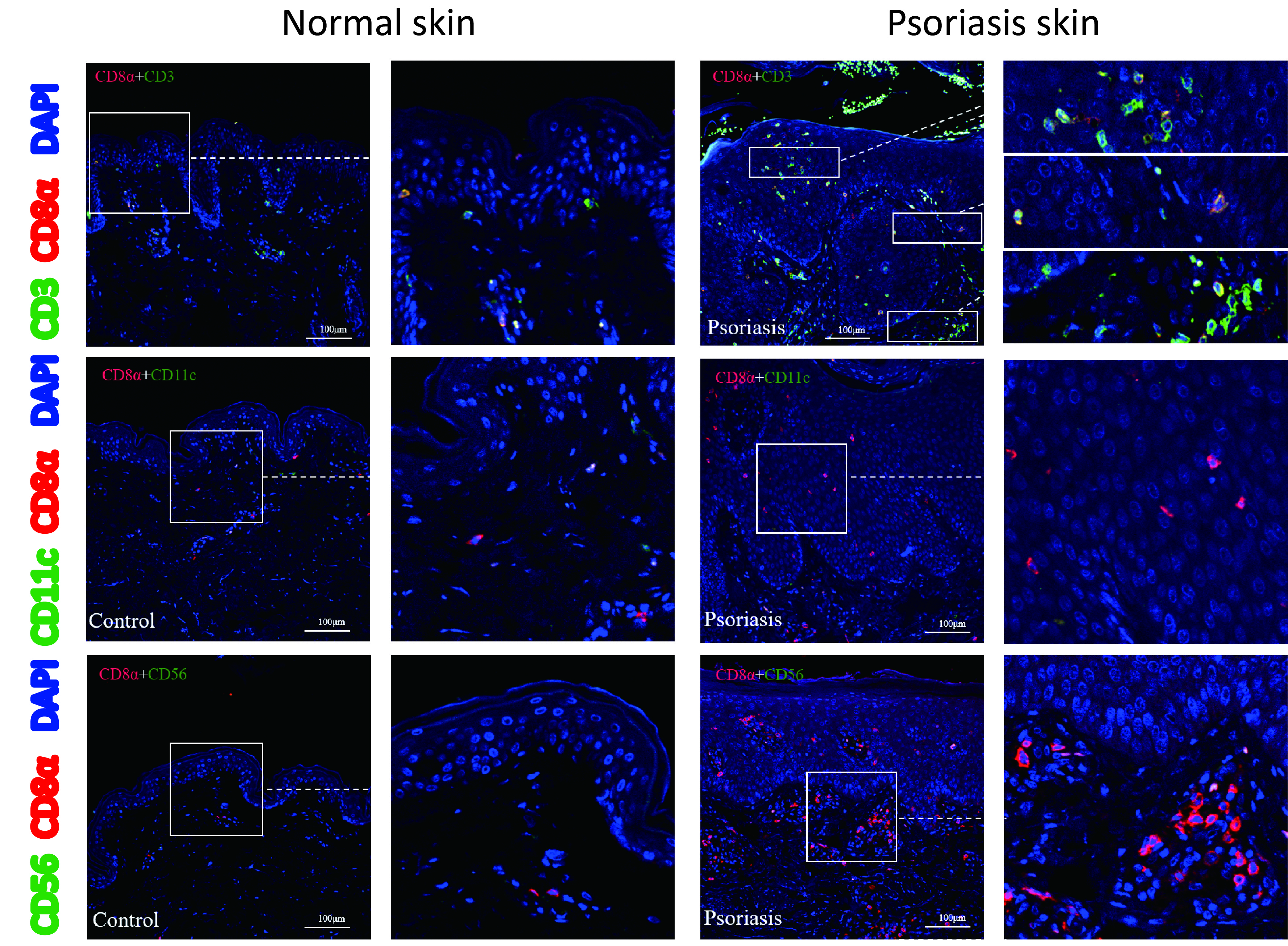

Supplement: Supplementary file 2 — Supplementary Material 2 [file SKI2-1-e64-s002.jpg]

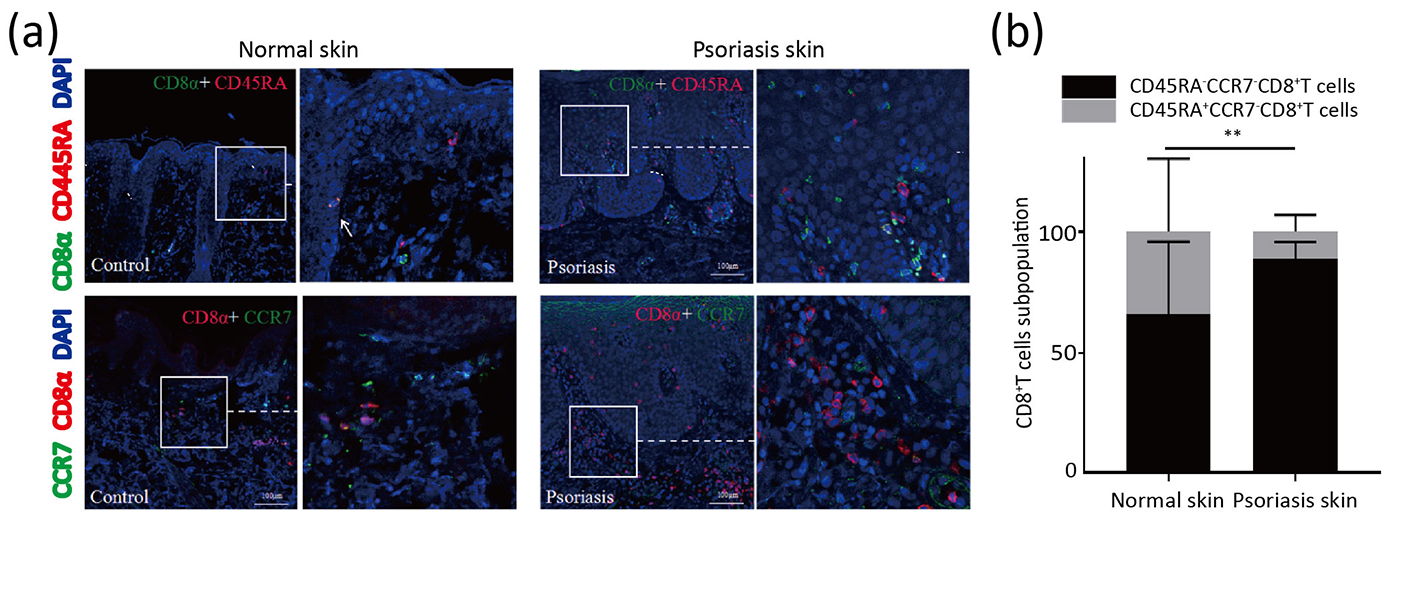

Supplement: Supplementary file 3 — Supplementary Material 3 [file SKI2-1-e64-s001.tif]
